# Digital and community-engaged approaches to improve research participant recruitment – Advice from forty convened experts

**DOI:** 10.1017/cts.2026.10720

**Published:** 2026-03-09

**Authors:** Loretta M. Byrne, Hailey N. Miller, Cyd Lacanienta, Cassie Lewis-Land, Daniel E. Ford, Cheryl R. Dennison Himmelfarb

**Affiliations:** 1 Institute for Clinical and Translational Research, Johns Hopkins University, Baltimore, USA; 2 Johns Hopkins University School of Nursing, USA; 3 Johns Hopkins Institute for Clinical and Translational Research, USA; 4 School of Medicine, Johns Hopkins University, USA

**Keywords:** Recruitment, retention, community engagement, social media, trust

## Abstract

Achieving enrollment goals is essential for the successful completion of a clinical trial. This includes enrolling a sample size that provides adequate power and engaging a study population that supports generalizability of research findings. Yet, trial participation is routinely hindered by its complexity, associated risks, and frequently cited barriers to participation including lack of awareness, low trust/mistrust, and logistical burdens that make participation of low value or unrealistic to potential participants [1,2].

## Introduction

Achieving enrollment goals is essential for the successful completion of a clinical trial. This includes enrolling a sample size that provides adequate power and engaging a study population that supports generalizability of research findings. Yet, trial participation is routinely hindered by its complexity, associated risks, and frequently cited barriers to participation including lack of awareness, low trust/mistrust, and logistical burdens that make participation of low value or unrealistic to potential participants [1,2].

The National Institutes of Health (NIH) and the National Center for Advancing Translational Sciences (NCATS) encourage the Clinical and Translational Science Award (CTSA) consortium to share effective strategies for recruiting clinical research participants. In response, the Johns Hopkins Institute for Clinical and Translational Research (ICTR) hosted a two-day event in Baltimore in the fall of 2024, titled “Digital and Community Engaged Approaches to Support Research Recruitment.” The goal of the event was to create a venue to discuss promising practices to improve participation in research. Through Linked-In, Facebook, ResearchMatch liaisons, and the CTSA’s Trail Innovation Network’s comprehensive list of sixty-seven institutional points of contact, invitations were sent. Each recipient was encouraged to disseminate the invitation to their researchers, research teams, recruitment and community engagement specialists, community partners, and students. In addition, community engagement presenters were encouraged to invite a community partner to co-present and share their tools, strategies, and insights on community-engaged approaches. The event provided a compendium of strategies that can be customized for local implementation by other academic research institutions. This summary highlights key presentations with details in Supplemental material, Appendix A.

### Overview of Event

A short talk format (7–15 minutes) allowed forty speakers to provide overviews of current recruitment practices to mitigate barriers to participation. Two hundred and twenty recruitment professionals, students, research coordinators, and local and regional community partners, representing the CTSA coalition (38/64 institutions) (East coast, 62%; Midwest, 16%; South, 12%; West coast 10%), attended with 88% attending both days. Registration was $350; lower for poster presenters and waived for speakers and invited community members.

Speakers highlighted that research teams need to work with recruitment specialists, frequently based in CTSA programs to enhance their study recruitment and that successful recruitment requires utilization of many different platforms. The keynote speakers delivered calls to action for the improved development and utilization of resources to support participant recruitment. Table 1 synthesizes their primary suggestions. Recordings and slides are available on the Johns Hopkins’ ICTR webpage: https://ictr.johnshopkins.edu/service/recruitment/.

**Table 1.** Top keynote speaker suggestions: digital and community engaged approaches to support research recruitment conference

1	Listen to and include the community’s needs when designing studies and institutional priorities.
2	Commit to enrollment targets, ensuring study population demographics reflect the disease’s prevalence.
3	Build trust in biomedical research by involving a robust network of community stakeholders throughout the research process.
4	Accelerate institutional funding and regulatory mechanisms so community partners can become meaningfully engaged as partners, faster.
5	Combine digital and non-digital recruitment strategies to adapt to different circumstances (e.g., using the EHR for cohort identification but messaging in person or with postal mail).
6	Ensure recruitment materials emphasize why participation is important to the participant (e.g., community impact or direct benefit).
7	Test the recruitment effectiveness of study messaging and imagery across different settings and populations and publish these findings.
8	Improve the simplicity and transparency of informed consent documents.
9	Compensate participants; it is fair and just.
10	Share study results with the community with clear explanations of how the data could be used.

## Day 1: National digital recruitment approaches

### Overview

Day one presenters discussed recruitment methods using social media posts/advertisements, research registries, and electronic patient portals. Given that studies show that many people may participate in research if they are asked and informed [3,4], digital methods of recruitment seem an ideal medium, yet the speakers acknowledged that digital strategies require access to and interest in social media, online registries, email and text messaging, and electronic health record digital platforms.

## Promoting clinical trials using social media

Four speakers described usage of the social media platform Meta (Facebook, Instagram). To increase views by those likely to meet inclusion criteria, speakers stressed that researchers should tailor campaigns for age, health condition, and geographic area with concise messages (50-character limit), utilizing a carousel of images or videos rather than static advertisements. They noted that precise control over who sees posts/advertisements is limited by platform algorithms but emphasized targeting posts toward viewers who may share characteristics (e.g., interests, age ranges, etc.) to those actively engaged as study participants, and that this strategy had improved post reach.

To determine effectiveness of creative elements, the CTSA’s services piloted study posts and advertisements on Facebook pages they managed using modest budgets ($100 over 1 week) and tracked performance using industry benchmarks ($1 per click and a 1% click-through rate), and metrics like candidate screener completion and enrollments. Effectiveness of campaigns varies by study specifics [5], but speakers mentioned study accrual ranging from $330 to $1,200 per enrollment and average budgets for social media at $2,000 to $5,000.

Overall, their effective social media recruitment strategies required posts using understandable and concise language, thoughtful imagery, and data from their pilot testing of a posts impressions, views and click-rates, to determine timeline and budget, along with informative landing pages and eligibility screeners detailing the study, contact information, and providing direction [6–8].

## Advancements in registry projects

Research recruitment registries, which serve as lists of individuals amenable to being contacted by researchers about participation in research, offer opportunities to improve recruitment for multiple projects. Whether disease specific or agnostic, registries must also recruit and retain participants [9–11]. For example, Vanderbilt University Medical Center (VUMC) described increasing enrollment into its disease agnostic registry, ResearchMatch.org, through advertisement on its patient MyChart dashboard with guidance from patient stakeholders, and an advisory council. As a result, ten percent of patients visiting the registry from MyChart self-registered within the first few months.

The All of Us Program employed a non-digital tactic to provide information and dialogue. The “All of Us Journey,” used a mobile van to provide space for education and bi-directional conversations in local communities. They reported that this aided in recruiting underrepresented individuals in biomedical research.

Some teams layer additional communication or services onto the traditional registry format to establish long-term engagement. For example, Johns Hopkins University’s (JHU), CONNECT platform connects individuals to cardiovascular health related research and delivers information via text messaging about heart health and research participation. The team is assessing how text messaging, its frequency, tone, timing, and content can build awareness of, trust in, and willingness to participate in clinical trials [12].

## Advances in electronic health record (EHR) recruitment services

EHR based recruitment can be a resource for identifying and sharing research opportunities with potentially eligible participants through patient-portals such as MyChart. JHU speakers reported that recruitment messaging through their patient portal has an average response rate of 5–10%, that opt-out rates from research messaging invitations are low (<1%) and most message recipients agreed that continued contact is a good use of MyChart [13,14]. A common concern of this recruitment method is dissatisfaction from patients. The speakers emphasized the importance of a governance structure/council to assist in evaluating the wording and frequency of messages and finding the best ways to share research opportunities to prevent this method from eliciting negative reaction from patients. Also discussed was the need to be respectful of the sensitive nature of messaging via a clinical medium. Presenters emphasized the need for clear communication along with measures to prevent patient message fatigue by setting monthly contact limits for outreach to patients and daily maximums for study team outreach.

A general feeling among presenters was that broadcast capabilities are enhanced when an institution moves from an “opt-in” to “opt-out” institution (patients who once had to opt-in to be contacted about research, now must opt-out). For example, the Medical University of South Carolina described changing to an opt-out institution. Since then, eighty-four research teams have used their EHR recruitment service; 30,476 patients contacted, with 0.31% of patients opting out [15].

Some EHR recruitment services have added new demographic choices in their EHR allowing patients to share language preference, sexual orientation, gender identity, and disability status to help focus their recruitment messaging. Speakers also reported using enrollment goals for race, ethnicity, and sex characteristics to guide message distribution strategies adjusting with each wave of messages to prioritize balanced representation in enrollment. Presenters recognized the challenge of identifying the best strategy for messaging patients via EHR-based recruitment methods. To address this, a decision matrix tool has been developed to guide research teams in selecting which EHR strategies may be most effective given a trial’s characteristics such as the inclusion criteria, and the site’s capabilities [16].

## Fostering trust by returning study results

Disseminating the results of research studies with the public and those that participated in the research is a promoted best practice for research and may foster trust in research and researchers, a critical barrier to participation [17,18]. The All of Us (AoU) program discussed returning specific information that is valued by the AoU participants, including individual genetic results [19].

The Recruitment Innovation Center (RIC) suggested planning for the dissemination of results early, providing participants with opportunities to learn the results of the study in multiple formats, and that null results are important to share [20]. The RIC shared infographics, narratives, and case studies (Supplemental material, Appendix A).

Lastly, VUMC’s ResearchMatch.org registry platform, which lists manuscripts associated with its use, leveraged ChatGPT-4 to summarize the abstracts of these manuscripts, in hopes of improving readability for a lay audience [21].

## Day 2: National community engagement approaches

### Overview

Day two highlighted practices and methodologies in initiating and sustaining community engagement that offer an array of options for consideration by researchers designing recruitment approaches. Presenters provided local strategies for involving community partners and members in planning throughout the research life cycle in efforts to make research participant-centered and community-informed.

## Best practices: Community partners in research planning and design

Engaging community partners in recruitment planning was emphasized by presenters as instrumental to address the underlying barriers of lack of awareness and lack of trust in research commonly experienced in local communities (e.g., plain language messaging, utilization of a community advisory board). The Community Engagement Studio (CES) model, described by VUMC and Meharry Medical College, has been widely adopted by research institutions. CES provides structured opportunities for investigators to receive feedback from community members and lived experience experts at all stages of the research process. The CES process offers practical modifications to study design and implementation. The speaker emphasized lessons learned from the program include that all lived experiences are valid and varied and may run counter to existing stereotypes or societal narratives. Researchers using this model report improved recruitment and reduced participant attrition after acting on community advice [22].

At the Children’s Hospital of Philadelphia, the Research Family Partners Program engaged parents, caregivers, siblings, and community members to advise pediatric researchers. This resource enables practitioners and researchers to collaborate with trained family representatives. Underscored was the long-term value of family and community advisory committees in pediatric research, including:

- Creating a supportive and feasible study plan for participants
- Finding and retaining participants
- Understanding and overcoming barriers to research, enhancing diversity of subjects
- Identifying and returning results that positively impact the community [23]

Johns Hopkins’ Community Research Advisory Council described their community engagement training project that provides early-career investigators with opportunities to learn plain language and patient-centered communication skills directly from community members [24]. To further mitigate barriers to participation, Centro Sol, a JHU center that consults and instructs on best practices for engaging with Latino communities, described options to foster culturally responsive research partnerships [25].

As a multi-state enterprise, Mayo Clinic discussed how their researchers have support from the CES model, five community advisory boards (Minnesota, Arizona, Florida, Rural Health, and Healthy Nations) and multiple community partnerships. The institution works toward the harmonization of resources across the institution, citing the development of a one-stop-shop to find community engagement support and citing examples that have increased the budget and duration of awards [26].

## Becoming worthy of community trust: Community engagement as a pathway to improve participation in research

These speakers highlighted customized approaches and utilization of community advisors to develop interventions and sustain engagement among local Asian American, Latino, Native American, and African American communities [27]. The presenters encouraged adoption of tailored strategies aligned with community needs (as shared by advisors) to foster inclusive engagement. The presenters utilized community advisory groups to help their teams facilitate meaningful connections, promote awareness, and provide valuable insights into community perceptions and barriers to participation. They provided examples of local advisory group objectives, key activities, and timelines [28,29].

George Washington University with others is exploring new ways to apply proven approaches. Among their projects, Community Health Workers (CHW) who are sources of science-based health information particularly within Spanish-speaking communities are disseminating research information via social media. The program seeks to demystify research while addressing the motivations and concerns of community members, such as the consequences of trial participation if uninsured [30].

Examples of how institutions can support researchers seeking to collaborate with rural reservation-based communities were presented and discussed a research project aimed at reducing suicide rates and supporting the safe transition from adolescence to adulthood among Native American women [31]. The speaker emphasized the importance of understanding the high-risk context for those living on a reservation and tribal sovereignty obligations. She stressed empowering Native American youth to pursue careers in healthcare and science, as professionals from indigenous communities influence development of innovative health care reforms that respond to the needs of communities they serve, and that researchers must engage participants at all stages of the research process to foster trust building and address a long-term commitment to the community’s defined needs [32].

Morgan State University shared an institutional approach for sustainable community-academic partnerships. The network is described as a hub (Morgan CARES) for community and academic researchers to connect and form long-lasting partnerships through grassroots funding, expert consultation, project management and access to facilities [33].

## Best practices: Community partners in research implementation

The final session emphasized maintaining community-academic partnerships [34]. The experts suggested these relationship-building practices:

- Communicate openly about institutional constraints, share firsthand experiences, and define each partner’s intentions.
- Share knowledge: actively train and share resources.
- Assess compatibility and capacity: do not assume every Community-Based Organization (CBO) is suitable or possesses the necessary resources.
- Establish points of contact and collaborate beyond research projects.
- Respect shared expertise: value the skills of community members, include them as key personnel or investigators.
- Honor the CBO’s time, processes, and prior commitments.
- Promote the CBO’s participation in meetings and decision-making processes.

In this way, the University of Southern California built relationships with CBOs then implemented a CHW program (*Promotoras de Salud*). One hundred and ninety-four Promotoras received 12 hours of training on research fundamentals, in Spanish, and were compensated with a $60.00 gift card and certification for their time. Notably, 46% of the participants in this program referred someone to a research study, and 39% of the Promotoras were more likely to participate in studies [35,36].

Similarly, the University of Texas Southwestern’s speaker described the multi-site HealthStreet program’s efforts to address local barriers to recruitment including mistrust, fear, lack of knowledge, misconceptions, historical experiences, and racism. The program integrated bilingual CHWs to enhance communication within their areas, by including research education with their health resource-sharing practices (Kahalnik, et al., 2025, unpublished manuscript). They reported that at their locations, this program makes discussions about research a component of community interaction [37].

In response to the need for information about COVID-19 vaccines, the *iHeard* program was launched as a multi-institutional initiative intent on disseminating accurate health information specifically at a local level where misinformation is being heard (https://nihceal.org/resources/promoting-health-knowledge-through-community-partnerships). Through short intake questionnaires, the program captured people’s perceptions on health issues, identified top information gaps, and delivered evidence-based messaging via social media, printed materials, and community engagement methods [38]. Finally, the Baltimore CONNECT initiative (https://www.bmoreconnect.org) described helping JHU researchers through traveling resource fairs bringing essential services into communities while facilitating researchers’ engagement capacity [39].

## Conclusion

Day one reinforced that thoughtful collaboration between researchers, data informatics teams, and community partners is key to improving broad population recruitment and strengthening trust in research processes. Day two underscored the importance of community-informed practices and tailoring approaches to local contexts and communities being served, with key community informants’ involvement. Finally, these strategies may address the recurring barriers noted in research participation, including lack of trust, accessibility and understanding of science.

Throughout, the speakers noted these strategies for more effective recruitment:

- One-size-fits-all recruitment does not work.
- Digital platforms provide opportunities for tailored recruitment approaches often at acceptable cost.
- Strategies improve when adapted to local context and study goals.
- Co-design with representative community advisors yields the strongest tactics.

Common themes for further work centered on:

- Institutions should support researchers in crafting respectful, understandable messaging and training recruiters to communicate in community-preferred ways.
- Institutions need to support researchers in demonstrating trustworthiness (early authentic engagement; minimizing barriers to participation; returning results to participants and community members).
- Evaluation of recruitment strategies will strengthen the field.

Of the conference’s evaluation responders, 64% were very satisfied with the event. The three most highly rated sessions were on social media, returning results and becoming worthy of community trust. Some reported that the costs of registration and in-person attendance were prohibitive and limited accessibility to people who were self-paying. The event was not promoted to the public and may have benefited from being more inclusive. Despite some limitations, the event highlighted practices that demonstrate promise for improved research recruitment and sustained community engagement across different environments. It is hoped that research teams, along with their institutional support systems, will recognize that their recruitment challenges mirror those faced within the CTSA consortium. By learning of successful strategies, and sharing across institutions, our goal is that others will emulate and expand on lessons learned, create new tools, and provide training and access to institutionally supported resources so that research teams may test these methods and publish their experiences, expanding the field of understanding. This includes access to institutionally supported social media pages for pilot testing and the launching of ads and posts, access to registries and EHR recruitment methods, access to training or support for crafting effective recruitment messages as outlined in the presentations, and avenues for collaboration with community engagement specialists, community members, and community organizations. Finally, this event highlighted the importance of combining recruitment strategies with community-engaged research approaches that support sustained partnerships throughout the research life cycle.

## Supporting information

10.1017/cts.2026.10720.sm001 Byrne et al. supplementary material Byrne et al. supplementary material

## Supplementary material

The supplementary material for this article can be found at https://doi.org/10.1017/cts.2026.10720.
