# Supplementary material for: Digital and community-engaged approaches to improve research participant recruitment – Advice from forty convened experts
Source: J Clin Transl Sci. 2026 Mar 9;10(1):e62. doi: 10.1017/cts.2026.10720 (PMC13107068; doi:10.1017/cts.2026.10720)
Supplement: Byrne et al. supplementary material [file S2059866126107201sup001.docx]

| **Appendix A. Speakers, Institutional or Organizational Affiliations, Talk Titles, Practice Point(s)** | |
| --- | --- |
| **Day 1** | |
| **Speaker, Affiliation, Talk Title** | **Practice Point(s)** |
| **Promoting Clinical Trials Using Social Media** | |
| Cody Gardner, MBA; University of Rochester; *From Impressions to Enrolls: 4 Years of Data-Driven Recruitment with Facebook Ads at the University of Rochester* | Data driven approaches:  • Utilize pilot campaigns.  • Prioritize video content (slideshows made with tools like Canva.  • Employ targeted ads to balance enrollment (create separate, targeted ads to ensure balanced enrollment). |
| Brooke Crockett-Nemeth, BA; The Ohio State University; H*arnessing Meta Ads for Effective Clinical Trial Recruitment* | Navigating Meta's evolving restrictions:  • Stay current with Meta's ad policy changes.  • Optimize using visuals that match study criteria  • Ensure ads are concise (use questions or short statements). |
| Ruth Gebrezghi, BA; University of California San Francisco; *Finding Your Participants on Social Media* | Strategies for effective recruitment:  • Customize strategy to diverse populations (vary the platforms for audiences like YouTube, TikTok, and Reddit)  • Focus on optimized ad graphics ( use infographics, assure contrast levels for readability)  • Work to mitigate fraud in screening tools (add Captcha verification, repeat questions with different wording) |
| Angela Fritsche, MPA; Mayo Clinic; *Creating and Implementing a Tiered Social Media Marketing Campaign for a Clinical Trial* | Approaches on all levels require focus on patient benefit more than research details:  • Match the marketing effort to the recruitment needs of study.  • Create integrated, multi-channel campaigns (paid search, socials, native ads, TV)  • Use custom marketing landing page (tracks metrics and conversions). |
| **Keynote Discourse: Innovations in Digital Recruitment** | |
| Cheryl Dennison Himmelfarb, PhD, RN: Vice Dean for Research; Sarah E. Allison Endowed Professor, Johns Hopkins School of Nursing; Deputy Director, Johns Hopkins Institute for Clinical and Translational Research | |
| Stephen Juraschek, MD, PhD, FAHA, AHSCP-CHS: Associate Professor of Medicine, Harvard Medical School; Assistant Professor of Nutrition, Harvard T.H. Chan School of Public Health; Research Director, Hypertension Center at Healthcare Associates; Associate Director for Ambulatory and Community-Based Research  Division of General Medicine, Section for Research, Beth Israel Deaconess Medical Center | |
| Scott D. Halpern, MD, PhD: University of Pennsylvania: John M. Eisenberg Professor of Medicine, Epidemiology, and Medical Ethics & Health Policy; Director, Palliative and Advanced Illness Research (PAIR) Center; Director, Behavioral Economics to Transform Trial Enrollment Representativeness (BETTER) Center, University of Pennsylvania Perelman School of Medicine | |
| **Advancements in Registry Projects** | |
| Lisa Connally, BS, CCRP; University of Michigan; *Utilizing Social Media Advertising in Conjunction with an Engaged Volunteer Registry to Raise Awareness about Research Opportunities at the University of Michigan and Beyond* | • Social media posts and ads early on led to 20,814 new volunteers joining registry. • Registry model is available and has expanded to Chicago and Miami |
| Hailey Miller, PhD, RN; Johns Hopkins University; *From Research Registry to Online Community: Enhancing Heart Health and Research Participation Through Engagement and Education* | • Synergizing community-based and digital methods for recruitment and engagement is essential for overcoming known barriers to recruitment  •Tailoring outreach and research opportunities by participants is important in the design of research registries and communities  • Team is testing a model where participants also receive education on heart health and research participation as part of registry model |
| Sarah Cook, MPH; Recruitment Innovation Center; *Innovation through information: Recent updates to the ResearchMatch platform* | • Advertising on VUMC's Epic MyChart dashboard led to 10% of patients registering on ResearchMatch from the MyChart link. • Registry is integrated with Epic at VUMC. Account creation is assisted through transfer of information. • Volunteers can provide Expert Advice and share opinions on study designs. |
| Minnkyong Lee, PhD; NIH All of Us; *All of Us Journey:* *Meeting Communities Where They Are* | • 827,000 participants enrolled via community-engaged practices and CTSA collaborations. • Provided education and enrollment opportunities in the community via a mobile fleet of buses taking staff to 40+ events (2022-2023). • Fostered by a nationwide network of local community partners (health alliances, colleges, municipal organizations, etc.) |
| **Advancements Using Digital Approaches** | |
| Angela Fritsche, MPA; Mayo Clinic Comprehensive Cancer Center; *Creating and Implementing an Educational Clinical Trial Video Delivered Using the Electronic Patient Portal* | Patients are made aware of the importance of research via an educational video sent via patient portal 72 hours before their visit. |
| Fred Stevenson, RN, MSN, FNP, with Siobhan Vasquez, CCRP; University of California Davis Health; *Invitation Accepted: How UC Davis Health Delivers Secure Recruitment at Scale* | • UC Davis Health and Studypages developed a secure, data-driven mass email/text recruitment strategy leveraging media-consumption insights and cross-functional collaboration to deliver rapid, scalable, PHI-compliant outreach. • This methodology was implemented by innovative stakeholders in the CTSC, UC Legal, IRB, Compliance, and IT Cybersecurity, ensuring end-to-end PHI protection for an EMR cohort. |
| Jodie Cohen, MA, CCRP; Recruitment Innovation Center; *A Mobile Device URL IRL: Innovating Multisite Study Recruitment with the Clinician Study “App”* | • The RIC would like to present the Clinician Study App (CSA), a new mobile tool for multi-site studies that replaces traditional study information cards. • Clinicians get instant access to study details and site-specific contacts, thereby encouraging provider referrals. |
| Abiodun Otolorin, MD, MS, FAAFP; Howard University; *Digital Health Infrastructure for Health Equity Research* | • To address disparities in the D.C area, researchers ‘go into the community’ with an Interactive Digital Signage device, displaying study information.  • They are creating an MHealth mobile app to share trial details internally and externally. • They are developing a clinical data repository of deidentified clinical data for research use, to support cohort identification, and the creation of predictive models. |
| **Developing and Sustaining Electronic Health Record Recruitment Services** | |
| Sarah Nelson, MS; Recruitment Innovation Center; *Identifying EHR-driven Recruitment Strategies for Consultations in the Trial Innovation Network’s Recruitment Innovation Center* | Evidence-based EHR recruitment in multi-center trials: •Use Decision Guide (structured process for EHR strategies, tech specifications of site) •Create computable phenotype (logic rules specific for EHR query identifying candidates) •Tailor strategy to context (site-specific workflows, and technical resources) |
| Katherine Connors, MPH; Stanford University; *Stanford's Participant Engagement Platform (PEP):* *A Control Center for Epic MyChart, Direct Email, and Postal Mail Honest Broker Services* | Honest broker model for direct to patient recruitment services:  •Honest broker model protects patient privacy as study team does not see identified candidates until they express interest. •Create automated, scalable processes that prioritize diversity (data queries that optimize for race, ethnicity, and sex).  •Utilize secure, effective, and privacy-compliant EHR portals (Epic MYChart), along with direct email (for studies including controls), and postal mail. |
| Tara Pittman, MA, CCRP; Medical University of South Carolina; *Lessons Learned in MUSC’s First Years of Implementing an Opt-Out Approach to Contacting Patients Identified Through the Electronic Health Record* | Best practices for shift from institutional "opt-in" to an "opt-out" recruitment policy:  • Create multi-disciplinary governance committee (reviews process, workflows, and guidelines for study teams identifying potentially eligible subjects via the EHR).  • Create standardized documentation of patient contact and research contact preference across recruitment report delivery platforms (Epic Workbench, REDCap) and train teams on documentation before report release. |
| Molly Belinski, MPA; University of California San Francisco; *The UCSF EHR Recruitment Service: A Scalable Model that Prioritizes the Patient Experience* | Study team directed EHR recruitment:  •Highlight experience and privacy (set limits on number of messages per month).  •Create self-service EHR management platforms that study teams can utilize.  •Establish real-time dashboards for study teams to track enrollment metrics by population. |
| **Returning Results/Returning Value** | |
| Deidra Crews, MD, ScM, FASN, MACP; Johns Hopkins University; *Enhancing Trust in Research through Return of Results* | Perceived trustworthiness of research is influenced by: • Perceptions that researchers and institution are competent, benevolent, and have integrity • Research participant attributes (e.g. health status) • Contextual factors (e.g. public messaging or friend and family views of research) • Researchers demonstrating trustworthiness (early authentic engagement; minimizing barriers to participation; returning results to participants and community members) |
| Minnkyong Lee, PhD; NIH All of Us; *NIH All of Us Research Program: Advancing Genomic Research through Community Engagement* | • All of Us offers the return of individual genetic information to the participants as part of their efforts to return value and information back to participants. |
| Sarah Cook, MPH; Recruitment Innovation Center; *Disseminating Research Results to Participants and the Public* | Find clear examples and templates of how to return results (infographics, narratives, and case studies available on the Meharry-Vanderbilt Community Engaged Research Core website |
| Cathy Shyr, PhD; Recruitment Innovation Center; *Leveraging Artificial Intelligence to Generate Lay Research Summaries on a National Recruitment Platform* | • ResearchMatch program uses ChatGPT-4 to summarize published abstracts of the papers posted on the ResearchMatch Study Results page. • Summaries are at a literacy level accessible to the public.  • Prompts for ChatGPT4 prioritize succinctness, clarity, and practical relevance. |
| **Building Sustainable Partnerships with the IRB and the Community** | |
| Megan Singleton, JD, MBE, CIP; Johns Hopkins School of Medicine | Focus on how important it is to establish collaborations between researchers, Institutional Review Boards (IRB) and community partners: Partnerships can result in increased research process transparency, and Community Research Advisory boards that provide bi-directional input to research teams, and foster trust in communities. |
| Roger Clark, MBA; Johns Hopkins, Community Co-PI CEAL DMV Team; Community Co-Chair Community Research Advisory Council |  |
| Christopher Chute, MD, DrPH; Johns Hopkins School of Medicine |  |
| **Day 2** | |
| **Keynote Discourse: The Crucial Role that Community Engagement Plays Throughout the Research Process** | |
| George Mensah, MD, Director, The Center for Translation Research, and Implementation Science (CTRIS) at the National Heart, Lung, and Blood Institute | |
| Joni Rutter, PhD, Director, National Center for Advancing Translational Sciences (NCATS) at the National Institutes of Health (NIH) | |
| Cheryl Dennison Himmelfarb, PhD, RN: Vice Dean for Research; Sarah E. Allison Endowed Professor, Johns Hopkins School of Nursing; Deputy Director, Johns Hopkins Institute for Clinical and Translational Research | |
| **Best Practices: Community Partners in Research Planning and Design** | |
| Leslie Boone, MPH; Recruitment Innovation Center; *Meharry-Vanderbilt Community Engagement Studios: Learning from Lived Experiences to Inform Research Design and Implementation* | • Repeatedly asking the same population, the same questions appears disingenuous and community stakeholders feel researchers do not listen. • Communities and patients want to know what researchers changed because of their recommendations. • Responding to actionable recommendations provided, is expected. |
| Heather Cathrall, MBE; Children’s Hospital of Philadelphia; *Research Family Partners; Understanding Patient & Community Perspective: A System for Enabling Authentic Family& Community Engagement in Pediatric Health Research* | • Families serve as advisors, co-investigators, board members, and are even included in study publications as co-authors. • Families are personally onboarded and undergo training including (www.fyreworkstraining.com) • Fully cost recovered from grants except for one institutionally funded family panel to allow ALL researchers access to community feedback • Families compensated for their time. RFP Staff receive FTE funding typically 3-5% per project |
| Michael Thompson, BS; Johns Hopkins ICTR; *Community Research Advisory Council Governance* | • Community members to mentor trainees in community engagement. • The TL1, T32 School of Nursing, or K12 trainees develop the skills for explaining their research to a lay individual/community member. |
| Monica Guerrero Vazquez, MPH, MS; Johns Hopkins University; *Effective Engagement of Latinos in Research and Healthcare Through Collaborative Advocacy* | • Promote equitable practices i.e., participant compensation, transportation, childcare, healthcare access • Recruit bilingual AND bicultural study teams  • Collaborate with the IRB, submit culturally appropriate materials with your protocol • Design and implement tools that are accessible i.e. REDCap/Qualtrics Spanish modules, mobile distribution, text messages |
| Sagar Dugani, MD, PhD, MPH; Mayo Clinic Rochester; *MN Community Engagement Strategies for Academic Institutions* | • The Community Scientist Program builds community leadership in research by training community members on the research process who go on to have roles within research. • Mayo Clinic uses specific community advisory boards (CABs) that reflect different regions and populations (Rochester, MN; Jacksonville, Florida; Phoenix, AZ; Healthy Nations, Rural Health, and Pediatrics). • Assess the value of community-engaged research (CEnR) programs and developing new methods to adapt to needs and change. |
| **Becoming Worthy of Community Trust: Community Engagement as a Pathway to Participation in Research** | |
| HaeRa Han, PhD, RN, FAAN; Johns Hopkins University; *Reflecting on the Clinical Trial Journey with a Community Advisory Board in an Asian Immigrant Community* | • Johns Hopkins University study utilized a CAB to inform research design, culturally and linguistically tailored outreach and advocacy for participants with limited English proficiency as part of a multi-site clinical trial on dementia in Korean American older adults with undiagnosed dementia and their caregivers . • Materials were accessible, relevant, and respectful.  • Researcher shared CAB's key activities in different stage of the research cycle (formation, implementation, sustainability). |
| Elizabeth L Andrade, DrPH, MPH; The George Washington University; with Ivonne Rivera, MPH; *Something Old and Something New: The marriage of community health workers and digital technology to engage Latino communities in research* | • Presenters highlighted continuous shifts in community trust related to health sciences research.  • GWU shared promising strategies, including use of CHWs as primary implementers of interventions; use of private Facebook groups to establish rapport and connectivity, foster engagement with multimedia; increase digital health literacy in communities to support |
| Teresa Brockie, PHD, MSN, BSN, RN; Johns Hopkins University; with Deserae Kill Eagle, BS, MPA; Johns Hopkins University; *Becoming Worthy of Community Trust: Community Engagement as a Pathway to Health Equity and Diverse Participation in Research* | •Presenters provided the context that places Native American communities at high risk for suicide, particularly those living in reservations and rural areas.  •They shared the First Nations Data Governance Strategic Framework and socio-ecological factors that influence Native American youth suicide in Fort Belknap Indian reservation.  •Presenters described tribally-centered collaborating activities over a five-year period for their intervention study.  •They described a pipeline for future Indigenous practitioners in the STEM disciplines.  •Tribal leaders and community are the decision makers in tribal jurisdictions and control data and research. |
| Payam Sheikhattari, MD, MPH; Morgan State University; *Bridging Academia and Community: The Role of the Morgan Cares Network and Community Research Councils in Sustainable Collaboration* | • Morgan State University described the development of a hub (Morgan CARES) for community and academic researchers to connect and form long-lasting partnerships.  • Key functions of Morgan CARES were listed, with services that cover the research stages of partnership development, innovation, collaborative action and outcome and impact.  • Presenter described the roles and benefits of a community research council in enhancing representation of all communities in studies. |
| **Best Practices: Community Partners in Research Implementation** | |
| Nicole Wolfe, PhD; University of Southern California; *The Important Role of Community Health Workers/Promotoras de Salud in Research: The Research Ambassador Training Program* | • Building understanding of clinical research with the community surrounding USC in southern California was facilitated through an ambassador model between local Promotoras and USC researchers.  • Attitudes toward willingness and participation in research were measurable.  • 45.7% - Percent of participants in program referred someone to a research study |
| Farra Kahalnik, MPH, MSW; University of Texas Southwestern Medical Center; *HealthStreet: Reaching People Where They Live and Work to Provide Services, Build Trust, and Improve Diversity in Research Participation* | • Bilingual Community Health Workers facilitate  communication and trust between local community and research teams. • Provides free health screens in community settings for underrepresented racial and ethnic groups • Links community members to care, resources, and Community Research Registry |
| Jennifer C. Erves, PhD, MPH, MAEd, MS, CHES; Vanderbilt University Medical Center; *Advancing Community-Academic Partnerships through Bidirectional Engagement and Partnership Equity* | • External factors and inputs may impact power differentials. • Develop a shared language. • Advocacy may be needed for each partner to engage equitably in the research process. |
| Alexis Marsh, LMSW; Washington University in St. Louis and Donald Young: Johns Hopkins Institute for Clinical and Translational Research; *iHeard’s Community Engaged Research* | • To improve health knowledge and foster trust in science, the institutions partnered with 'hyper local' community to assess and address gaps in knowledge and mitigate the spread of inaccurate health information. • Systematic mobile phone surveys useful • Rapid responses to prioritized local, timely topics • Share-ready print and social media messages sent weekly to trusted messengers |
| Patricia Barger, BA, MBA; Executive Director, and Veena Thamilselvan, MSPH: Baltimore CONNECT Inc.; *Community-Driven Partnerships with CEnR Teams Bring Resources and Reliable Information to Baltimore Residents* | • Organized traveling resource fairs facilitate connection across the community and the local institution.  • Ask for support and involvement from local elected officials • Provide food, make it fun, and accessible via public transportation • Block funding agreements between institutions and community partners allow for agile responses to changing community needs • Radical transparency based in high level information sharing and level setting supports CBPR relationships |
